# Using a Bayesian approach with reverse philosophy to design clinical trials in rare diseases

**DOI:** 10.1186/1745-6215-14-S1-O58

**Published:** 2013-11-29

**Authors:** Lucinda Billingham, Emma Hall, Clare Cruickshank, Jim Barber, Steve Nicholson

**Affiliations:** 1 MRC Midland Hub for Trials Methodology Research and Cancer Research UK Clinical Trials Unit, University of Birmingham, Birmingham, UK; 2 Institute of Cancer Research Clinical Trials and Statistics Unit, University of London, London, UK; 3 Velindre Hospital, Cardiff, UK; 4 Charing Cross / Chelsea and Westminster Hospital, London, UK

## 

A standard approach to trial design is to test a null hypothesis of no treatment effect in terms of a primary outcome measure against an alternative hypothesis of a minimal clinically relevant treatment effect as chosen by the investigators. Sample size is determined to maximise the chance that the trial detects this effect if it exists whilst minimising the chance of a false positive conclusion. We propose a reverse philosophy is used in rare diseases where the design starts with the number of patients that is feasible to collect within a sensible time frame and then, based on a Bayesian analysis, show that this amount of data could provide useful information on which to make clinical decisions in the future.

This paper illustrates application of this approach to design the first ever potentially practice-changing trial in penile cancer, as part of the International Rare Cancers Initiative. The primary outcome measure is survival time with treatment effect measured as a hazard ratio. Given a predicted number of events, the design is evaluated by (i) demonstrating the information that the trial could provide for a range of possible observed results and prior distributions; and (ii) given a pre-specified decision criteria, using simulation to determine the probability that the trial will make the correct decision under different underlying true scenarios.

Treatment decisions in rare diseases should be based on trial evidence but the traditional approach to design is problematic and using a Bayesian approach with reverse philosophy may provide a practical alternative.

